# EnaS: a new software for neural population analysis in large scale spiking networks

**DOI:** 10.1186/1471-2202-14-S1-P57

**Published:** 2013-07-08

**Authors:** Hassan Nasser, Selim Kraria, Bruno Cessac

**Affiliations:** 1 Neuromathcomp, INRIA/UNSA, Sophia-Antipolis, France; 2 Dream, INRIA, Sophia-Antipolis, France

## 

With the advent of new Multi-Electrode Arrays techniques (MEA), the simultaneous recording of the activity up to hundreds of neurons over a dense configuration supplies today a critical database to unravel the role of specific neural assemblies. Thus, the analysis of spike trains obtained from in vivo or in vitro experimental data requires suitable statistical models and computational tools.

The EnaS software [7], developed by our team, offers new computational methods of spike train statistics, based on Gibbs distributions (in its more general sense, including, but not limited, to the Maximal Entropy - MaxEnt) and taking into account time constraints in neural networks (such as memory effects). It also offers several statistical model choices, some of these models already used in the community (such GLM [6] and the conditional intensity models [5]), and some others developed by us ([1] and [2]), and allows a quantitative comparison between these models. It also offers a control of finite-size sampling effects inherent to empirical statistics.

EnaS allows large scale simulation thanks to our recent study [2] (hundreds of neurons) with spatio-temporal constraints. It's available as a Graphical User Interface in order to make the tools more accessible by non-programmers. Within EnaS framework, programmers are also allowed to implement new tools and integrate them with the existing modules. We featured EnaS with parallel processing on personal computers (using MPI) and on clusters (Using OpenMP).

**Figure 1:**
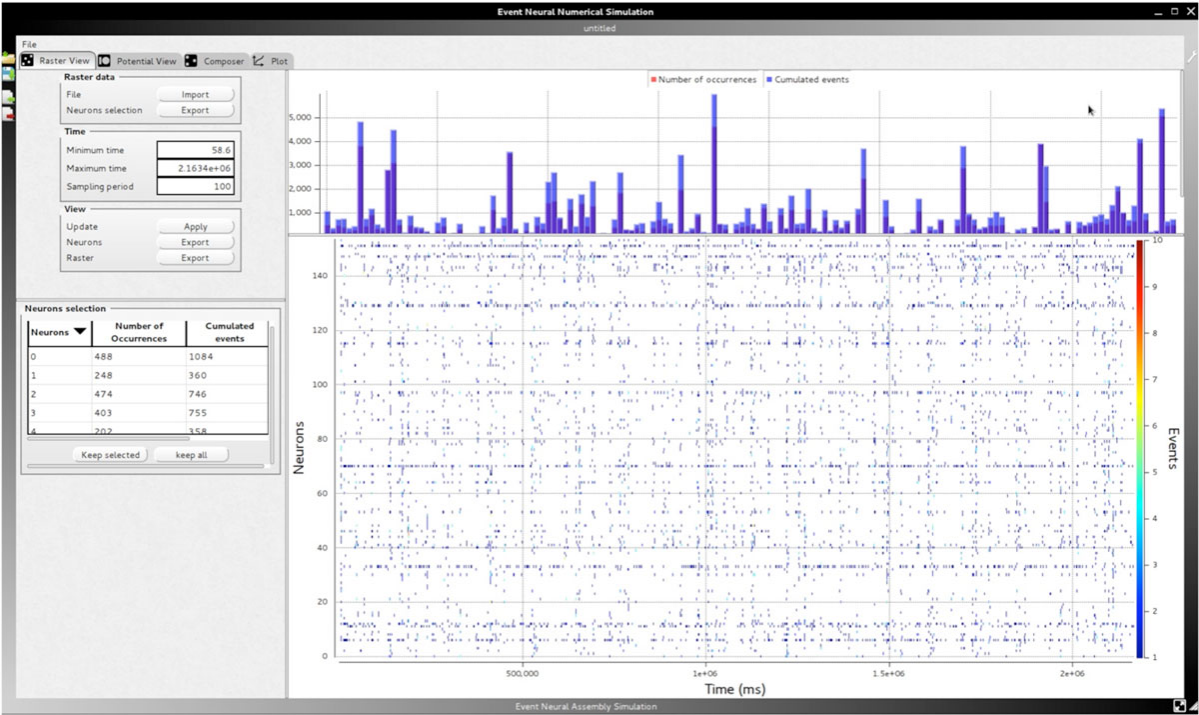
**The GUI of EnaS**. This page allows displaying a spike-train, showing the firing rates and configuring the binning value (sampling rate) of the data. It also allows selecting a subset of neurons and sorting the neurons with respect to their activity.
